# Visual attention to faces during attractiveness and dominance judgements

**DOI:** 10.1017/ehs.2025.2

**Published:** 2025-02-14

**Authors:** Žaneta Pátková, Vít Třebický, Martin Kocourek, Dagmar Schwambergová, Karel Kleisner, Jan Havlíček, Jitka Třebická Fialová

**Affiliations:** 1 Faculty of Science, Charles University, Prague, Czech Republic; 2 Faculty of Physical Education and Sport, Charles University, Prague, Czech Republic; 3 Center for Virtual Reality Research in Mental Health and Neuroscience, Third Faculty of Medicine, Charles University, Prague, Czech Republic; 4 National Institute of Mental Health, Klecany, Czech Republic

**Keywords:** Mate choice, sexual selection, attractiveness, dominance, competition

## Abstract

Perception studies describe numerous discrete morphological facial features as important to judgements of various characteristics. Interestingly, little is known about whether people actually direct their visual attention to these features and how specific contexts or sex affect this attention. We, therefore, examined visual attention to faces in the context of intersexual (opposite-sex assessment of attractiveness) and intrasexual (same-sex assessment of dominance) selection.

In total, 93 women and 33 men rated 80 high-resolution facial photographs of men and women while their gaze was recorded using eye-tracking. To explore patterns of raters’ attention to faces and specific facial features, we used the number of fixations, fixation duration, and visit duration as visual attention measures.

Women directed more visual attention towards the faces of potential partners (more fixations) than potential rivals, and men had longer fixation duration when assessing potential partners than rivals. Facial features that acquired the most visual attention across contexts and sexes were the eyes, nose, and mouth, but small differences between the sexes and contexts in visual attention were found for other facial regions suggested by previous perception studies, such as the chin and the cheeks indicating their importance in specific judgements.

## Graphical abstract

**Figure:**
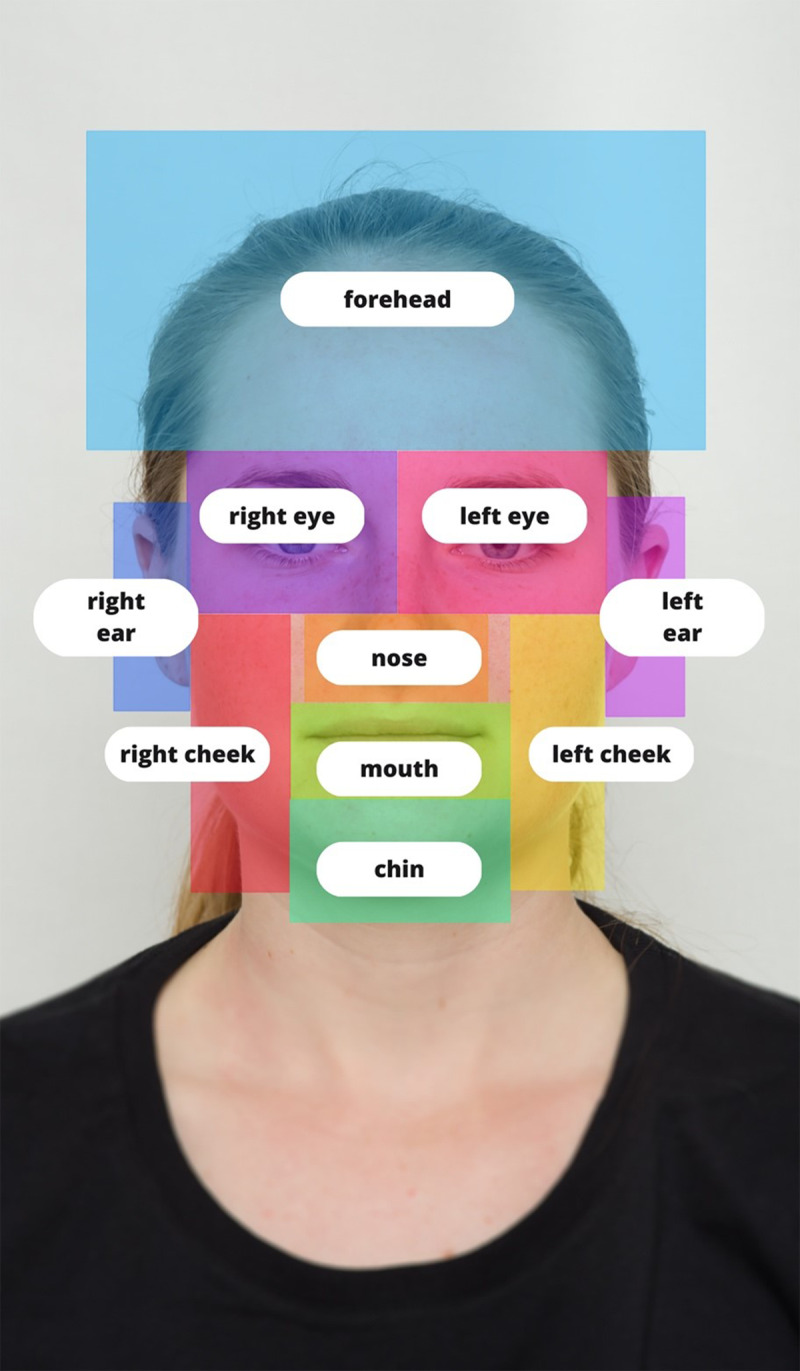

## Social media summary

Eye-tracking shows eyes, nose, and mouth draw the most attention in facial attractiveness and dominance judgements.

## 1. Introduction

People are exceptionally attentive to the faces of others (Gillath et al., 2017; Hewig et al., 2008) and spontaneously attribute many characteristics, for instance age, sex, attractiveness, and personality including dominance (Calder et al., 2011; Little, 2014; Perrett et al., 1998) based on facial appearance. These assessments are usually formed rapidly and with just thin slices of available information, such as the variation in development of certain facial features.

The ability to adequately assess the characteristics of others based on their appearance may be crucial for making decisions about own future actions. In the context of mate choice, such decisions might be about the suitability of a potential partner (for review, see Havlíček et al., 2022; Thornhill & Gangestad, 1999, 2006). In the context of competition for mates, it can include a decision about whether one should compete with a potential rival or withdraw (Sell et al., 2012). One may therefore expect selection for neurocognitive mechanisms that facilitate adequate perception, judgement, and behaviour (Galperin et al., 2013).

Intersexual and intrasexual selection are considered to be significant selective pressures which lead to the development of certain traits in humans (Třebický et al., 2012). It has been suggested that in the context of intersexual selection, the attractiveness of certain traits functions as a cue to the individual’s mating quality, such as health, quality immune system, or developmental stability (Stephen & Luoto, 2023). The tendency to be attracted by individuals who have attractive traits is believed to increase own fitness via direct or indirect benefits rising from potential mating with such individuals (Thornhill & Gangestad, 1999, 2006). However, recent studies often fail to find the expected links between facial attractiveness and various measures of health (Cai et al., 2019; Foo et al., 2017; Jones et al., 2021; Pátková et al., 2022). Regarding morphological facial traits influencing perceived attractiveness, studies generally show that higher facial symmetry (Rhodes et al., 2001; Little, 2014; but see Kleisner et al., 2024) and averageness (Kleisner et al., 2024; Little, 2014; Rhodes et al., 1999) are perceived as attractive. Sexual dimorphism also influences perceived attractiveness, with higher femininity generally considered attractive in women (Fiala et al., 2021; Perrett et al., 1998; Rhodes et al., 2003). However, research on facial masculinity in men shows mixed results (Burriss et al., 2014; Fiala et al., 2021; Perrett et al., 1998; Rhodes, 2006). As for specific size and shape of individual facial features, morphological studies show that in women, features such as large eyes, small noses, fuller lips, and rather gracile chins are considered attractive (Abend et al., 2015; Cunningham, 1986; Pflüger et al., 2012; Schaefer et al., 2006), while in men, it is, e.g., large eyes, fuller lips, smaller noses, and prominent cheekbones and chin (Cunningham et al., 1990; Windhager et al., 2011).

Analogically, intrasexual selection in men is thought to shape morphological traits connected to both perceived and actual formidability, aggressiveness, dominance, or other traits related to success in competition in general (Barber, 1995; Puts, 2010). Intrasexual selection in women received less attention, and some authors argue that women compete with each other mainly in terms of attractiveness (Fink et al., 2014; Fisher, 2004). This is primarily due to a lower incidence of overt physical aggression among women compared to men (Knight et al., 1996, 2002). However, under specific ecological and cultural circumstances, women may participate in overt physical aggression in the context of mate acquisition and retention (Ness, 2004; Rosvall, 2011). It has been found that men and women perceived as more dominant have more masculine features, such as smaller eyes, thin lips, wider cheekbones, prominent brow ridge, robust jawline, and narrow lips (Keating, 1985; Třebický et al., 2013; Vernon et al., 2014; Windhager et al., 2011). Nevertheless, recent research highlighted methodological considerations regarding the association between facial masculinity and dominance. Positive associations are often found when using computer-manipulated stimuli in a two-alternative forced choice (2AFC) but not when employing unmanipulated stimuli in sequential presentation (Dong et al., 2023). Although it has been suggested that certain facial features are linked to the perception of attractiveness and dominance, it has not yet been directly investigated whether individuals actually selectively focus on these features when assessing faces.

Eye-tracking provides insights into autonomous visual attention processes. It also enables avoiding potential bias connected with self-reports, which can be affected by participants’ beliefs, including social desirability. Eye-tracking can identify the direction of visual attention through delineated areas of interest (AOIs), the number and duration of fixations (areas where the gaze rests), saccades (quick eye movements from one visual target to another), dwell time in the AOI (time spent looking at the area), or, similarly, overall visit duration in the AOI, which is a sum of all visits in the AOI (time between the first fixation on the AOI and the next fixation outside of the AOI) including saccadic duration between those fixations, which distinguishes it from dwell time. Various studies suggest that these metrics refer to different but sometimes also overlapping aspects of cognitive processes behind visual attention (Althoff & Cohen, 1999; Duchowski, 2017; Skaramagkas et al., 2023). It has been proposed that the higher number of fixations and longer overall visit duration could reflect the importance of areas where the gaze is directed (Jacob & Karn, 2003), correspond to the informativeness of visual stimuli and their liking, or indicate cognitive load (Duchowski, 2017; Skaramagkas et al., 2023). The duration of fixation is believed to be positively associated with task difficulty (Galley et al., 2015). Given that there can be a few long fixations or numerous short fixations, which can translate to the same overall visit duration in the AOI, examining the number and duration of particular fixations alongside visit duration might provide deeper understanding of gaze behaviour.

Previous studies using eye-tracking have provided information about the general AOIs when looking at people. It has been shown that both heterosexual men and women are most interested (measured in viewing duration) in the faces of opposite-sex individuals (Hewig et al., 2008). Further, heterosexuals look longer and more often at faces of the opposite sex who are potential partners than at the faces of potential friends (Gillath et al., 2017), which supports the notion of the face being especially salient in the mating context. Some studies also examined visual attention towards masculine and feminine faces and found that women looked longer and more often on feminised male faces in 2AFC test (Burriss et al., 2014), while other study showed there is no attentional bias towards task-irrelevant masculinised male faces in various experimental paradigms (Albert et al., 2023).

Eye-tracking studies have also investigated the specific facial regions in which people are generally interested. When freely looking at faces without a specific task (free-viewing paradigm), the eyes seem to draw the most visual attention, followed by either the mouth or the nose in the second place (Hickman et al., 2010; Król & Król, 2019; Semmelmann & Weigelt, 2018). Similarly, during the face recognition task, participants fixated the longest on the eyes, nose, mouth, and cheeks (Chelnokova & Laeng, 2011). Several studies examined possible differences in visual attention to facial regions in the context of specific judgements. During facial attractiveness judgements of women’s faces, both men and women looked the longest at the nose and then, for similarly long times, at the eyes and the lips (Zhang et al., 2017) and no sex difference in visual attention was found. A major limitation of that study was that the eye, nose, and mouth were the only AOIs analysed (Zhang et al., 2017). In studies examining a wider array of AOIs and comparing differences in visual attention in two assessment contexts (Hermens et al., 2018; Kwart et al., 2012), no differences in visual attention were found when judging the age and attractiveness of the face; participants fixated primarily on the eyes and nose (Kwart et al., 2012). Similarly, during trustworthiness and dominance judgements, visual attention was comparable during both tasks, with the eyes, the nose, and the mouth attracting the greatest amount of visual attention (Hermens et al., 2018). For further reading, see, e.g., Leder et al. (2016, 2010), Maner et al. (2008), or Mitrovic et al. (2020, 2016).

All in all, although mate choice and competition are considered to function as significant selective pressures linked to the development of specific facial features, little is known about the respective context-dependent differences in visual attention to faces and attention to specific facial regions. While some eye-tracking studies have tested changes in visual attention to faces between different contexts (Hermens et al., 2018; Kwart et al., 2012), to the best of our knowledge, direct differences between the context of mate choice and competition have not been studied yet. Moreover, the size of the stimuli in previous eye-tracking studies varied (Hermens et al., 2018; Kwart et al., 2012; Zhang et al., 2017), and where the stimuli were relatively small, it may have influenced the results due to the availability of parafoveal and peripheral vision (Hermens et al., 2018). On top of that, although perception and morphological studies (Cunningham et al., 1990; Mitteroecker et al., 2015; Schaefer et al., 2006; Třebický et al., 2013; Windhager et al., 2011) have identified several specific facial features relevant to attractiveness and dominance judgements (besides eye, nose, and mouth also, e.g., chin and cheeks), those features are not always specified as AOIs in relevant eye-tracking studies (Zhang et al., 2017).

**Figure 1.**
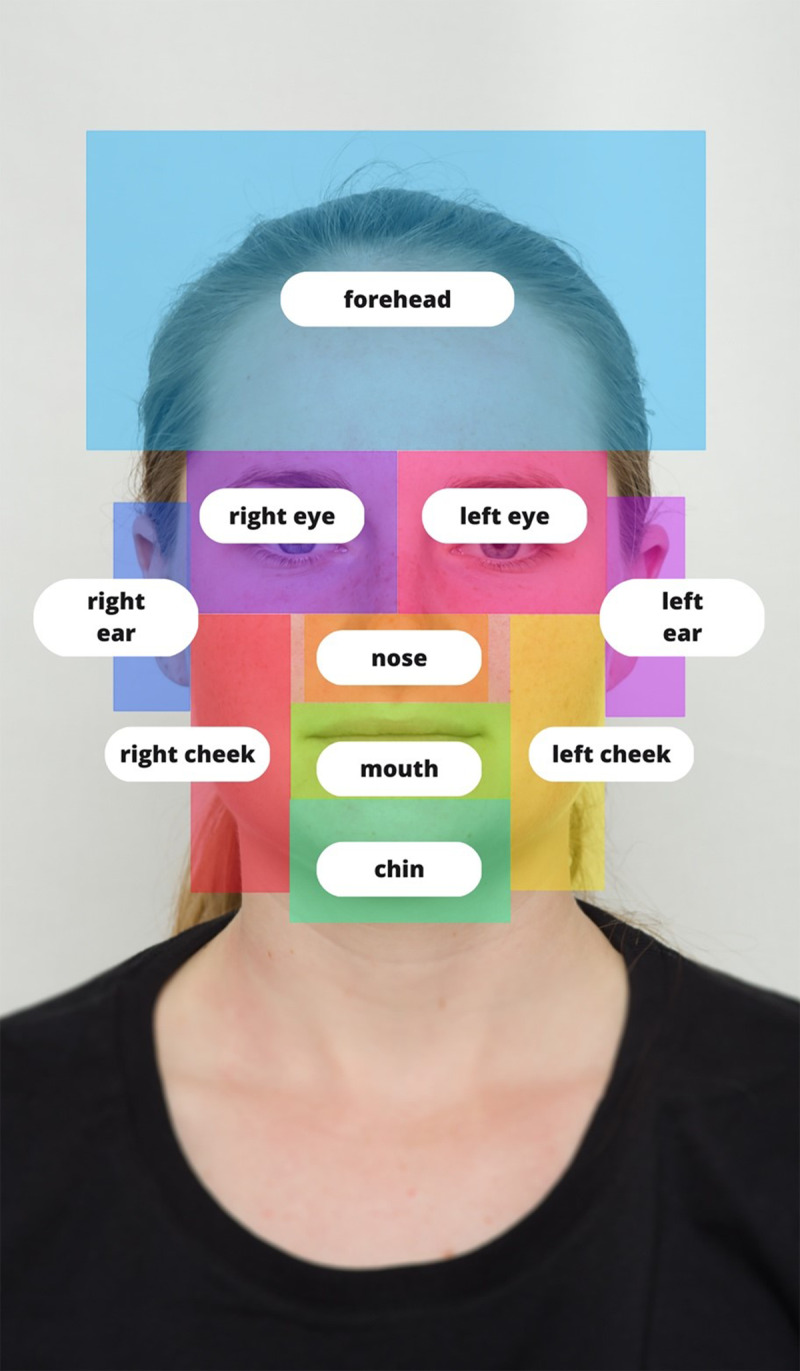
Example of stimuli with delineated AOIs (informed consent was obtained to publish the image).

In the present study, we investigate potential differences in visual attention to faces and their individual features in the context of mate choice (opposite-sex assessment of potential partners’ attractiveness) and competition (same-sex assessment of potential rivals’ dominance) using eye-tracking methods. To do that, we used close to life-size as possible, high-quality and high-resolution stimuli. We also specified multiple AOIs based on insights from previous studies, and besides eyes, nose, and mouth, we also included cheeks and chin (see Fig. 1), which have been shown to be relevant for judgements of attractiveness and dominance (e.g., Mitteroecker et al., 2015; Windhager et al., 2011). Moreover, we examine the possible effects of raters’ sex. As measures of visual attention, we used the number of fixations and overall visit duration as measures of the importance of the stimuli or its parts, fixation duration as a measure of the cognitive load the stimuli (or its parts) pose, and the AOI of the first fixation to investigate the importance of the specific AOI.

As previous studies showed that people look more at potential partners than friends (Gillath et al., 2017), we expected that men and women would pay more visual attention to potential partners (i.e., opposite sex) during rating of attractiveness than to potential rivals (i.e., same sex) during rating of dominance. For between-sex differences, we hypothesised that women would pay more visual attention to faces in both contexts than men would, as was also the case in Gillath et al. (2017). Based on the results of previous studies (Chelnokova & Laeng, 2011; Hermens et al., 2018), we anticipated that regardless of the context, men and women would direct most of their visual attention to the eyes, the nose, and the mouth. Further, we expected that both men and women would look more at features identified by perception studies (Třebický et al., 2013; Vernon et al., 2014; Windhager et al., 2011) as important in dominance judgements (e.g., chin) when assessing potential rivals than when assessing potential partners and that men would direct their attention to these features more than women, as studies suggest dominance judgements to be more relevant to men, attested by the higher incidence of overt physical aggression among them (Knight et al., 1996, 2002). Finally, we explored the place of the first fixations.

## 2. Materials and methods

The authors assert that all procedures contributing to this work comply with the ethical standards of the national and institutional committees on human experimentation and with the Helsinki Declaration of 1975, as revised in 2008. The study and its methods were approved by the IRB at Charles University (approval no. 2019/20).

Before entering the study, all participants were briefed into the data collection procedures and general scope of the study and expressed their consent with participation in the study by signing an informed consent form. Data used in this study are part of a larger longitudinal project investigating intra- and interindividual differences in visual attention to facial features which are believed to have developed under the influence of intrasexual and intersexual selection.

### 2.1. Procedure

Raters, in randomised order, assessed sets of facial photographs of same-sex individuals (40) for their dominance, and facial photographs of opposite-sex individuals (40) for their attractiveness on 7-point scales based on a situation induced by a short vignette (potential partner or rival). At the same time, their eye movements were recorded by eye-tracking. Participants then completed a set of questionnaires (e.g., regarding basic demographical data).

### 2.2. Raters

Raters were recruited via social media sites (Facebook), oral invitations, and posters in the halls of the Faculty of Science, Faculty of Humanities, and the Faculty of Physical Education and Sports (all Charles University, Prague, Czechia). Requirements for participation were age 18–40 years, being heterosexual, with normal or corrected-to-normal vision, and, in women, not being a user of hormonal contraception to avoid possible effect of hormonal contraception on their perception (Little et al., 2013). In total, 110 women and 35 men participated in the study. We excluded from further analyses 14 non-heterosexual women and 2 non-heterosexual men (defined as 3 and above on a 7-point scale ranging from 1 – exclusively heterosexual to 7 – exclusively homosexual). Further, data from three women were excluded due to insufficient quality of the eye-tracking data (where the eye-tracker did not identify the eyes correctly and fixations were either missing or on the side of the screen for most of the viewing session). The resulting sample thus consisted of 93 women (M = 23.5 years, SD = 4.37, age range = 18–38 years) and 33 men (M = 23.9 years, SD = 4.69, age range = 18–37 years). All raters received a reimbursement of 100 CZK (∼4 EUR) as compensation for their time (∼60 minutes).

### 2.3. Stimuli

The stimuli consisted of 80 standardised facial photographs of Czech men (40) and women (40) aged 19–34 years (men: M = 24.4 years, SD = 4.10, women: M = 23.3 years, SD = 4.25), a subset of photographs obtained in previous studies (Kleisner et al., 2019). We intended to keep the rating reasonably long and not too demanding for the participants. Photographs were selected based on their degree of standardisation. Targets were positioned 0.5 m from plain grey background and photographed from a distance of 1.5 m. They wore black T-shirts provided by researchers, assumed a neutral facial expression and refrained from any adornments such as glasses, jewellery, or makeup. Stimuli were captured with a Canon 6D full-frame DSLR equipped with an 85 mm fixed focal length lens under conditions standardised in terms of targets’ distance from the camera, environment, and exposure. For further details of the photo acquisition procedure, see Kleisner et al., (2019) and Třebický et al., (2018).

#### The post-processing of photographs

We used the Adobe Lightroom CC 2019 and Adobe Photoshop CC 2019 for the post-processing of photographs we had obtained. Images were colour calibrated with DNG colour calibration profiles (using the X-Rite Color Checker Passport Lightroom plugin). Evenness of exposure was manually checked and, where necessary, adjusted on the 85% value of every channel in the RGB colour space. Each participant’s horizontal and vertical position within the image frame was adjusted so that the target’s head was positioned in the centre of the frame with pupils on one horizontal line. Then we batch-cropped the photographs to optimally fit the heads on a 16:9 27″ monitor (resulting in head size slightly smaller than real life) while preserving the relative difference in size between individuals. In the next step, a blur vignette was applied over the photos so that the face, hair, and neck remained in focus, and mainly the T-shirts and surrounding parts of the background were slightly blurred to minimise any possibly disturbing creases or shadows. Then we converted the resulting images into an sRGB colour space and exported them into an 8-bit JPEG format (1215 × 2160 resolution, 300 PPI, sRGB). The stimuli occupied area equivalent to a visual angle of 12° (horizontal) by 21.4° (vertical) in size.

### 2.4. Eye-tracking

Rating was conducted using Tobii Studio software v 3.4.8 on a desktop computer with a 27″ LCD screen (BenQ PD2700U IPS; 3840 × 2160, 99% sRGB colour space coverage) in the landscape position. Eye-tracker Tobii X2-60 (60 Hz) was mounted to the bottom frame of the LCD monitor using a clamp and an extension arm. The eye-tracker was at a distance of 28 cm in front of the screen, tilted 13° upwards towards the participant and centred to the middle of the screen. The active width and height of the LCD screen to track were set to 60 × 34 cm, respectively. The upper edge of the eye-tracker was 4.5 cm above the lower edge of the LCD monitor.

### 2.5. Rating

Rating took place in a quiet windowless room under standardised conditions with artificial lighting so as to eliminate any changes in ambient light. The raters sat ∼90 cm from the screen with eyes at the height of 116 cm (i.e., at the same eye level as the stimuli on the screen when measured from the floor to the outer corner of the eye). Raters were seated on an office chair without wheels, with an adjustable headrest and armrests. Their head was resting against the headrest and arms against armrests, which were adjusted according to their needs. A large plastic pad was positioned in their lap: on the pad, they used a mouse to carry out the rating. Next, we performed a calibration of the eye-tracker using a standard 9-point calibration scheme in the Tobii software (Blais et al., 2008). If necessary, calibration was repeated. As soon as successful calibration was achieved, raters were instructed not to move or talk unless necessary. Then they carried out one testing round to familiarise themselves with the rating interface. During this trial, a smiley was shown instead of a facial photograph but other elements were the same as in the actual rating.

Each rater assessed both male and female sets of facial photographs, each containing 40 images. The two sets and photographs in them were presented in a randomised order. Participants assessed photographs of same-sex individuals regarding their dominance and photographs opposite-sex individuals regarding their attractiveness on 7-point scales. Before they started rating a set, we induced the context of potential partner or potential rival assessment by a short vignette (Csajbók et al., 2022). It was displayed in Czech on the screen. The vignettes had the following form (for men): ‘Imagine you are at a party. Suddenly, you notice that a woman standing nearby is looking at you with interest. How attractive is this woman according to you?’ or ‘Imagine you meet a woman at a party and spend a better part of the evening with her. Now, another man, who seems interested in her as well, approaches her. How dominant (i.e., how capable of enticing her away) do you think the man is?’ Analogous texts were displayed to women. Then a fixation cross was displayed for 1,000 ms in different quadrants of the screen (never in the centre of the screen, to avoid AOI fixation bias for the area where the stimuli were about to be presented) before each facial photograph and raters were instructed to always look at the fixation cross. This was followed by a 5,000 ms long presentation of the facial photograph. In the next step, a 7-point verbally anchored rating scale of attractiveness/dominance (atr/dom) was displayed for 7,000 ms on a new screen, where participants indicated their rating by clicking on the appropriate number.

After the rating session, raters completed questionnaires regarding their basic and demographic data (age, education, occupation, etc.), sexual orientation, in case of women also the phase of menstrual cycle, and other questionnaires unrelated to the current investigation.

The duration of viewing each facial photograph was set to 5,000 ms in the Tobii Studio software, and 5,000 ms filter was also set in jamovi for the visit duration. Aside from that, if a rater recognised the depicted in the photograph, that combination of rater and stimulus was removed from analyses (five raters in total recognised a minimum of two and maximum of five people in the dataset).

### 2.6. AOI delineation

In comparison to some previous studies which defined as AOIs only the eyes, the nose, and the mouth (Zhang et al., 2017), we defined other areas identified by perception studies as relevant for attractiveness and dominance judgements (Cunningham, 1986; Cunningham et al., 1990; Třebický et al., 2013), such as the cheeks, chin, and the forehead, similarly to Chelnokova and Laeng (2011). Using Tobii Studio software v 3.4.8, we have thus defined the following AOIs: right eye, left eye, nose, mouth, forehead (including hair), chin, right cheek, left cheek, right ear, and left ear manually for each stimulus. For an example of the defined AOIs, see Fig. 1.

## 3. Data analyses

All statistical analyses were performed in jamovi v 2.3.21.0. Inspection of the data parameters, normality tests (Kolmogorov–Smirnov), and visual representation indicated that the data for fixation duration, visit duration, and the number of fixations on the AOIs do not follow a normal distribution but a negative binomial. Only the number of fixations on a whole face was normally distributed. To investigate the data which followed a negative binomial distribution, we employed generalised mixed-effects models, while for the normally distributed data, we used linear mixed-effects model using the GAMLj module (v 2.6.6) in jamovi. For the analyses, we merged AOIs with left and right dichotomies into one AOI. That means that, e.g., from left and right eye AOI was created (by summation) one AOI ‘eyes’. The patterns of results remain virtually unchanged after merging the AOIs.

To test the effect of context and rater’s sex on visual attention (dominance vs attractiveness ratings), we conducted both whole-face analyses and analyses for separate AOIs. We entered the number of fixations, mean fixation duration (ms), and visit duration (ms) into separate models as dependent variables. The context of rating (atr/dom), and in the case of AOI analyses also ID AOI were entered as fixed-effect predictors. To control for the variability of targets and raters, we entered the targets’ and raters’ IDs as random effects. This showed that the target ID had virtually no variance, leading to a singular fit. Therefore, we report all analyses without target ID as a random effect.

Example of a model entry for whole-face analysis: *N* fixations per face ∼ 1 + rater’s sex + atr/dom + rater’s sex:atr/dom + (1 | ID rater), and AOI analyses: *N* fixations per AOI ∼ 1 + atr/dom + ID AOI + rater’s sex + atr/dom:rater’s sex + atr/dom:ID AOI + ID AOI:rater’s sex + atr/dom:ID AOI:rater’s sex + (1 | ID rater). To test differences between pairs of predictor levels, we used a post hoc test with Holm correction for multiple comparisons. For linear mixed-effects models, we report the proportion of variance explained by the fixed effects without random effects with *R*^2^_M_, the proportion of variance explained by both the fixed and random effects with *R*^2^_C_, and the effect size using *β* with 95% CI. For generalised mixed-effects models, we report fixed-effect omnibus test results with *χ*^2^ and, in case of whole-face analyses, the effect size using *β* with 95% CI.

To identify the AOI of the first fixation, we used the chi-square test of association. For between-contexts differences, we specified AOIs as rows and context (atr/dom) as columns. For between-sex differences, we specified AOIs as rows and rater’s sex as columns. We report *χ*^2^ and the strength of association with Cramer’s *V*.

## 4. Results

### 4.1. Whole-face analyses

**Figure 2.**
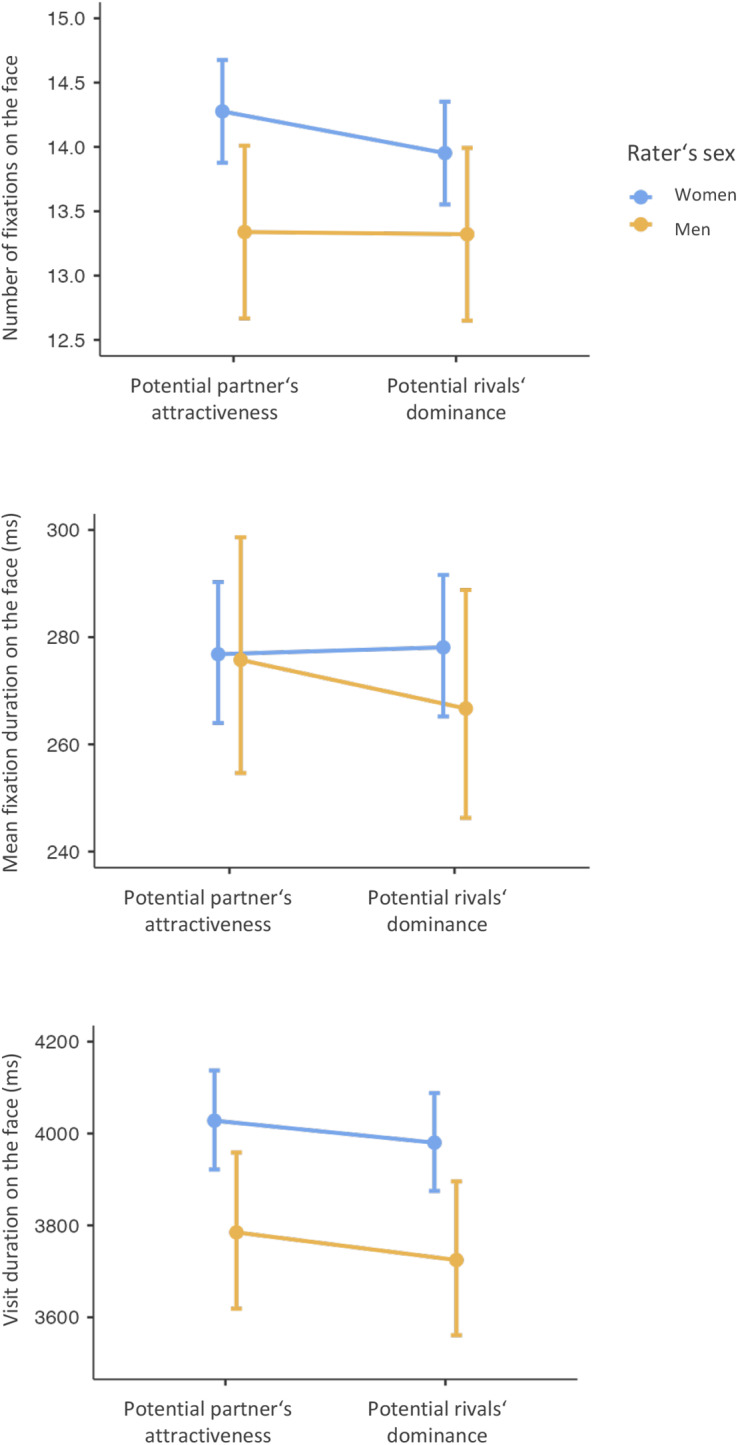
Context-related differences in visual attention in male (yellow) and female (blue) raters in whole-face analyses. From top to bottom: The number of fixations, mean fixation duration, and visit duration (in milliseconds). Dots represent mean values, error bars their 95% confidence intervals.

The linear mixed-effects model (*R*^2^_C_ = 0.408, *R*^2^_M_ = 0.015) showed that the *number of fixations* on the face was statistically significantly predicted by the rater’s sex (*F* (1, 123.993) = 4.021, *β* = −0.784, [−1.551, −0.018], *p* = 0.047), the context that is, by whether the rater was assessing a potential rival or a potential partner (*F* (1, 9940.003) = 10.328, *β* = −0.171, [−0.275, −0.067], *p* = 0.001) and by the interaction between rater’s sex and context (*F* (1, 9940.003) = 8.328, *β* = 0.307, [0.098, 0.515], *p* = 0.004). Women made, on average, statistically significantly more fixations (14.28 compared to 13.95) when assessing the attractiveness of a potential partner than when assessing the dominance of a potential rival. For details, see Fig. 2 and Supplementary materials S1 and S1J.

A generalised mixed-effects model showed that the *mean fixation duration* on the face was statistically significantly predicted by the context (*χ*^2^ (1) = 7.752, *β* = −0.014 [−0.025, −0.004], *p* = 0.005), but not by rater’s sex (*χ*^2^ (1) = 0.236, *β* = −0.023 [−0.115, 0.069], *p* = 0.627), and was predicted by the interaction between context and rater’s sex (*χ*^2^ (1) = 13.469, *β* = −0.038, [−0.058, −0.018], *p* < 0.001). Specifically, we found that in men, on average, the fixation duration is statistically significantly longer when assessing potential partners compared to rivals (276 ms compared to 267 ms). For details, see Fig. 2 and Supplementary materials S1 and S1J.

A generalised mixed-effects model showed that, face *visit duration* (time spent at whole face) was predicted by rater’s sex (*χ*^2^ (1) = 6.005, *β* = −0.064 [−0.116, −0.013], *p* = 0.014) and the context (*χ*^2^ (1) = 8.427, *β* = −0.014, [−0.023, −0.005], *p* = 0.004) but was not predicted by the interaction between rater’s sex and context (*χ*^2^ (1) = 0.179, *β* = −0.004, [−0.023, 0.015], *p* = 0.673). Women had statistically significantly longer visit duration on the faces during the rating (4,004 ms) than men (3,755 ms). Raters also had statistically significantly longer visit duration on the face during assessment of the potential partner’s attractiveness than potential rival’s dominance (3,905 ms vs 3,850 ms) For details, see Fig. 2 and Supplementary materials S1 and S1J.

### 4.2. AOI analyses

A generalised mixed-effects model showed that the *number of fixations* in AOIs was predicted by the context (*χ*^2^ (1) = 58.235, *p* < 0.001), by the AOI (*χ*^2^ (6) = 103,119.377, *p* < 0.001), by rater’s sex (*χ*^2^ (1) = 4.603, *p* = 0.032), by the interaction between context and AOI (*χ*^2^ (6) = 63.562, *p* < 0.001), by the interaction between context and rater’s sex (*χ*^2^ (1) = 145.503, *p* < 0.001), by the interaction between AOI and rater’s sex (*χ*^2^ (6) = 644.261, *p* < 0.001) and by the interaction between context, AOI, and rater’s sex (*χ*^2^ (6) = 208.296, *p* < 0.001).

Looking at the differences between contexts, women made, on average, statistically significantly more fixations on cheeks when assessing potential partners’ attractiveness than when assessing potential rivals’ dominance (0.318 vs 0.214). Further, women made statistically significantly more fixations on the chin of potential partners than rivals (0.293 vs 0.125). Women also made statistically significantly more fixations on ears during the assessment of potential partners compared to the assessment of potential rivals (0.288 vs 0.209). Women also made statistically significantly more fixations on the forehead while assessing potential partners than potential rivals (0.752 vs 0.66). On the other hand, men made statistically significantly more fixations on the chin of potential rivals than partners (0.246 vs 0.141).

**Figure 3.**
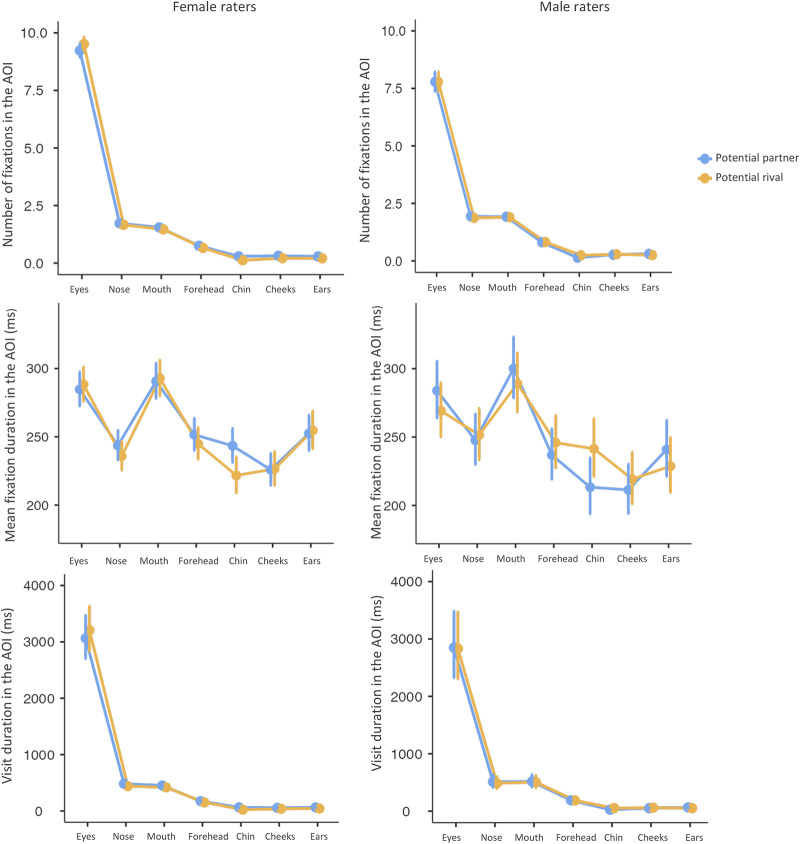
Differences in visual attention in AOI analyses. From top to bottom: the number of fixations, mean fixation duration, and visit duration (all with respect to particular AOIs). Potential partner’s attractiveness is marked in yellow and potential rival’s dominance is in blue. Female raters are on the left, male raters on the right. Dots represent mean values; error bars show their 95% confidence intervals.

For the differences between the sexes of the raters, women made statistically significantly more fixations on the chin when assessing potential partners than men (0.293 vs 0.141). Women also made statistically significantly more fixations in the eyes of potential partners than men did (9.231 vs 7.786) and they also made more fixations in the eyes of potential rivals than men (9.507 vs 7.785). Men made statistically significantly more fixations on the cheeks during the assessment of potential rivals than women (0.287 vs 0.214). Moreover, men made statistically significantly more fixations on the chin of potential rivals than women (0.246 vs 0.125). During the potential rival assessment, men made statistically significantly more fixations on the forehead than women (0.821 vs 0.66). During the assessment of potential partners, men made statistically significantly more fixations on the mouths than women (1.92 vs 1.55). During the assessment of potential rivals, men made statistically significantly more fixations on the mouth than women (1.9 vs 1.465). For details, see Fig. 3 and Supplementary materials S2 and S2J.

The generalised mixed-effects model had shown that *mean fixation duration* in AOIs was not predicted by the context (*χ*^2^ (1) = 0.316, p = 0.574), was not predicted by the rater’s sex (*χ*^2^ (1) = 0.315, *p* = 0.575), but was predicted by the AOI (*χ*^2^ (6) = 1304.241, *p* < 0.001), was predicted by the interaction between context and rater’s sex (*χ*^2^ (1) = 4.382, *p* = 0.036), was not predicted by the interaction between context and AOI (*χ*^2^ (6) = 5.728, *p* = 0.454), was predicted by the interaction between rater’s sex and AOI (*χ*^2^ (6) = 46.769, *p* < 0.001), and was predicted by the interaction between context, rater’s sex, and AOI (*χ*^2^ (6) = 47.427, *p* < 0.001). After inspection of the post hoc tests, there were no statistically significant differences in any of the comparisons of our interest. For details, see Fig. 3 and Supplementary materials S2 and S2J.

The generalised mixed-effects model showed that, the *visit duration* in AOIs was predicted by the context (*χ*^2^ (1) = 19.034, *p* < 0.001), by the AOI (*χ*^2^ (6) = 13,001.447, *p* < 0.001), by rater’s sex (*χ*^2^ (1) = 0.698, *p* < 0.001), by the interaction between context and AOI (*χ*^2^ (6) = 15.666, *p* = 0.016), by the interaction between context and rater’s sex (*χ*^2^ (1) = 53.477, *p* < 0.001), by the interaction between AOI and rater’s sex (*χ*^2^ (6) = 16.867, *p* = 0.010), and by the interaction between context, AOI, and rater’s sex (*χ*^2^ (6) = 147.741, *p* < 0.001).

Looking at the differences between contexts, women had statistically significantly longer visit duration on the cheeks of potential partners than rivals (57 vs 38.4 ms). Moreover, women had statistically significantly longer visit duration on the chin of potential partners than rivals (64.6 vs 24.1 ms). Lastly, women had statistically significantly longer visit duration on ears during the assessment of potential partners than rivals (63.8 vs 44.1 ms). Men had statistically significantly longer visit duration on the chin of potential rivals than partners (53 vs 26.5 ms).

For the differences between the sexes of the raters, women had statistically significantly longer visit duration on the chin during the rating of potential partner than men (64.6 vs 26.5 ms). Men had statistically significantly longer visit duration on the cheeks of potential rivals than women had (60 vs 38.4 ms), and men had statistically significantly longer visit duration on the chin of potential rivals than women (53 vs 24.1 ms). For details, see Fig. 3 and Supplementary materials S2 and S2J

In women, chi-square test showed no statistically significant difference in the *area of first fixation* between contexts: *χ*^2^ (6, *N* = 7396) = 4.047, *p* = 0.670, Cramer’s *V* = 0.02. Regardless of the context, the areas most frequently fixated as the first were the eyes, the nose, the forehead, and the mouth. For details, see Supplementary materials S3 and S3J.

In men, the chi-square test showed a statistically significant difference in the area of the first fixation between contexts: *χ*^2^ (6, *N* = 2628) = 19.377, *p* = 0.004, Cramer’s *V* = 0.086. Regardless of the context, the areas most frequently fixated as the first were the eyes, the nose, the mouth, and the forehead. When rating a potential partner, the first fixation was directed more frequently than expected (if it were by chance) to the nose, forehead, and cheeks than during rating of a potential rival. When rating a potential rival, men fixated first more often than expected on the eyes, mouth, and chin than during rating of a potential partner. For details, see Supplementary materials S3 and S3J.

Using the chi-square test of association, we found a significant difference between the sexes in the *area of first fixation* during assessment of potential partners: *χ*^2^ (6, *N* = 5017) = 63.789, *p* < 0.001, Cramer’s *V* = 0.112. The area most frequently fixated as first in both sexes was the eyes, the nose, the mouth, and the forehead. When assessing potential partners, women’s first fixation aimed more often than expected on the eyes, while men’s first fixation aimed more often than expected at the nose, the mouth, and the forehead. For details, see Supplementary materials S3 and S3J.

Using the chi-square test of association, we found a significant difference between the sexes in the area of the first fixation in assessments of potential rivals: *χ*^2^ (6, *N* = 5007) = 72.680, *p* < 0.001, Cramer’s *V* = 0.120. In both sexes, the most frequent first fixated areas were the eyes, the nose, the mouth, and the forehead. In assessments of potential rivals, women’s first fixation aimed more often than expected at the eyes, the nose, and the cheeks, while men’s first fixation aimed more often than expected at the mouth, the forehead, and the chin. For details, see Supplementary materials S3 and S3J.

## 5. Discussion

The primary aim of this study was to investigate potential differences in visual attention – measured by the number of fixations, fixation duration, and visit duration – towards faces and their features, using a suite of eye-tracking methods in the context of mate choice (attractiveness ratings of opposite-sex potential partners) and competition (dominance ratings of same-sex potential rivals). We investigated possible between-sex differences in visual attention in the two contexts using close-to-life-size, high-quality stimuli. When it comes to the whole face, women had marginally more fixations on the face when assessing potential partners’ attractiveness than when assessing potential rivals’ dominance, while men had longer mean fixation duration when looking at the faces of potential partners than the faces of potential rivals. To examine the importance of different facial features in the assessments, we have investigated the interest in particular AOIs identified based on previous research of facial perception. In both of the contexts described above, both men and women looked the most at the eyes, the nose, and the mouth, while the other areas (e.g., chin, cheeks) attracted little direct visual attention. In both of the analysed contexts, women made statistically significantly more fixations on the eyes than men did, while men made more fixations on the mouth than women did. In line with our expectations, we found variations in visual attention between contexts and sexes for features suggested by perception studies such as cheeks and chin.

Several previous studies provided some insight into contextual differences in visual attention to faces (Hermens et al., 2018; Kwart et al., 2012; Nguyen et al., 2009), but no direct comparison between mate choice and competition has been undertaken as yet. As noted above, we found that women made more fixations on faces when assessing potential partners than when assessing potential rivals. This suggests that women may be more interested in assessing opposite-sex potential partners’ attractiveness rather than same-sex rivals’ dominance. This is in line with the findings of Gillath et al. (2017), where men and women paid more visual attention to faces of potential partners than to the faces of friends, and with another study which showed that heterosexual individuals are interested in the faces of opposite-sex individuals more than in same-sex faces (Hewig et al., 2008). Further, no statistically significant differences in visual attention to whole faces between the two contexts were found for men, except for a marginally longer mean fixation duration when assessing potential partners. According to Galley et al. (2015), the lengthening of fixation duration signifies attention and cognitive control, which might be the case also in our study.

Regarding the particular facial features which attracted attention, our results are in line with previous eye-tracking studies (Hermens et al., 2018; Kwart et al., 2012) and show that, regardless of the context and rater’s sex, areas which attract the most visual attention are the eyes, the nose, and the mouth. This contrasts with a number of perception studies which indicated the importance of certain other facial features for judgements of attractiveness and dominance, for instance, besides the eyes, the nose, and the lips, also the chin and cheeks (Cunningham, 1986; Pátková et al., 2025; Scott et al., 2013; Windhager et al., 2011) and with those studies which suggested that faces are recognised rather by parts with the need to direct one’s gaze to those parts for detailed processing (Martelli et al., 2005). On the other hand, our results also should not be interpreted as implying that features other than the eyes, the nose, and the mouth play no role in attractiveness or dominance judgements at all. Eye-tracking shows where the gaze is directed specifically, and although we used stimuli as close to life size as possible, it does not mean that the raters did not have the remaining features in their field of vision. In other words, although the gaze was directed at, for instance, the eye, other features would have been still visible and could have contributed to the judgement. Moreover, the heightened interest in the eyes, the nose, and the mouth could be guided not only by interest in those facial features which are most conspicuous and/or ornamented but also by the assessment of intentions (via the direction of the gaze of the target, possible vocalisation, but also recognition of facial expressions), which is important in the formation of the first impression and, in our evolutionary past, would have been essential for avoiding costly mistakes in appropriate judgements (Kleisner & Saribay, 2019).

Although the eyes, the nose, and the mouth attracted by far the most visual attention, we detected small but statistically significant differences between contexts in visual attention to some other facial features as well. For instance, women paid more visual attention (measured as a number of fixations and total visit duration) to the chin and cheeks when assessing potential partners than when assessing potential rivals, which might point towards their importance in attractiveness judgements. This is in line with the proposed importance of the eyes, cheekbones, and chin for judgements of male attractiveness (Cunningham et al., 1990). On the other hand, the chin also seems to be salient for dominance judgements in previous morphological studies, mainly in men (Třebický et al., 2013). This is supported by our finding that men had more fixations and longer total visit duration on the chin when assessing potential rivals than when assessing potential partners. Therefore, while for women the chin was more important in the mating context than in rivalry, for men it was the opposite. Our results thus suggest that for men the chin plays a more important role in male intrasexual competition (Keating, 1985; Vernon et al., 2014) than mate choice. It is also possible that chin is a more salient feature in male faces in general as both men and women used it (had more fixations and longer total visit duration) for their respective assessments. Lastly, women also paid attention to the forehead and ears of potential partners more than rivals, possibly indicating interest in more facial areas during the potential partner rating.

We have observed sex differences in visual attention to AOIs in the two rating contexts. Women made more fixations at the eye region than men did during both tasks. This is in line with the study by Hall and colleagues (2010), which focused on recognition of facial expression and suggested that women are better at it thanks to paying more attention to the eyes. In this study, we used facial photographs of individuals with a neutral expression but still were able to detect in women increased visual attention to the eye area. Further, when assessing potential partners, women exhibited more fixations and longer visit duration on the chin than men did. This may be due to the importance of facial features such as jawline in attractiveness judgements (Cunningham et al., 1990; Little et al., 2011), connected, for example, with judgements of masculinity (Windhager et al., 2011). In contrast, men, when assessing potential partners, exhibited more fixations on the mouth than women did, which may indicate attention to potentially attractive and neotenous features in which the appearance of the lips plays an important role (Cunningham, 1986; Keating, 1985). When assessing potential rivals, men exhibited more fixations and longer visit duration on the cheeks, the chin, and more fixations on the forehead and mouth. Mouth and chin have been previously identified as important in dominance judgements (Rhodes, 2006; Scott et al., 2013); our findings thus provide further support to their relevance. The forehead isn’t commonly widely associated with either of attractiveness or dominance judgements, but Windhager et al. (2011) showed forehead shape changes in relation to an individual’s strength.

Lastly, the strength of our study lies in simulating a face-to-face viewing experience by the acquisition and presentation of the stimuli. Neither of the relevant studies used screens of comparable size and resolution (Hermens et al., 2018; Kwart et al., 2012; Nguyen et al., 2009; Zhang et al., 2017). Further, when comparing visual angles occupied by the stimuli, ours (12° by 21.4°) was larger than that of Zhang et al. (2017) and matched Hermens et al. (2018) in their large image condition (13° by 23°), which also aimed mimicking face-to-face viewing. Some other studies don’t provide this information (Nguyen et al., 2009) or are unclear in reporting (Kwart et al., 2012).

### 5.1. Limitations

There are several limitations to our study. Firstly, exploring the visual attention to faces in the context of mate choice and competition in heterosexuals, guided by our framework of intersexual and intrasexual selection, is necessarily linked with the sexes of the rater and stimuli. We didn’t opt for individuals to do same-sex assessment of potential partner’s attractiveness and opposite-sex assessment of potential rival’s dominance, given that we used vignettes to promote immersion in the task. If we take the example of male raters to illustrate it more clearly, we think it was not relevant for heterosexual men to rate other men as potential partners or rate women as potential rivals, enticing away their romantic interests. However, we acknowledge that our design confounds the effect of context (attractiveness of potential partner vs dominance of potential rival) and the sex (the effect of rater’s sex vs sex of the stimuli). This is a possible inquiry for future studies, which might help to disentangle further the effects of rating contexts and the effect of the sex of raters and stimuli. In this regard, future studies might avoid using our vignettes for same-sex attractiveness and opposite-sex dominance assessments. However, this poses a challenge; when men rate other men’s attractiveness, we can’t be sure what reasoning is behind this rating – isn’t it, e.g., an assessment of a potential rival in mate choice (intrasexual selection)?” One of the ways how to overcome this might be employing a control group where individuals would be rated in the friendship context; however, even this approach has its potential drawbacks, as studies show that men might often consider female friends as potential partners (Bleske & Buss, 2000).

Another potentially limiting factor might be a disbalance in our rater sample with fewer male than female raters. Still, both our male and female sample sizes were larger or at least comparable to many similar previous eye-tracking studies (Yang et al., 2015; Zhang et al., 2017). Another limiting factor might be the relatively low mean age of both the raters and the individuals who posed as stimuli (both were mostly young university students) giving limited insight into possible patterns in the general population. On the other hand, the match in age of rates and stimuli can be seen as an advantage of our sample as participants were assessing potential mates and rivals of roughly the same age.

### 5.2. Future directions

Future research in this area should investigate the same-sex assessment of attractiveness and opposite-sex assessment of dominance in addition to opposite-sex assessment of attractiveness and same-sex assessment of dominance, and it should focus on including a control group. Moreover, the research can focus on investigating what role varying degrees of attractiveness and dominance play in visual attention towards faces and their features. Lastly, studies should measure not only which features attract visual attention but also whether their appearance affects attention.

## 6. Conclusion

Our study contributes to research into visual attention to faces by examining it in two evolutionarily relevant contexts, namely assessment of potential partners (attractiveness rating) and potential rivals (dominance rating) and investigating sex differences in visual attention. Further, we used nearly-life-sized, high-quality stimuli and defined a wider array of theory-driven AOIs on the face than most studies do. We found contextual differences in visual attention to whole faces in women, who exhibited more fixations when assessing potential partners compared to rivals, pointing to the task’s importance or higher interest in it. At the same time, men had marginally longer mean fixations duration during the assessment of potential partners compared to rivals. Previous perception studies identified numerous morphological facial features as being important to judgements of attractiveness or dominance: besides the eyes, the nose, and the mouth also for instance the cheeks and the chin. Our study shows that the eyes, the nose, and the mouth are areas that indeed attract most visual attention across sexes and contexts. Nevertheless, in line with perception studies and our predictions, we have also found small differences in visual attention to the cheeks and the chin. For women, these features seem to be more important in the mating context, while for men, the chin seems to be a more salient source of information in male intrasexual competition, as attested by the fact women had longer visit duration and more fixations on chin when assessing potential partners and men when assessing potential rivals, respectively. Overall, our study suggests that visual processing of faces and attention towards individual facial features is to some extent both context- and sex-dependent.

## Research transparency and reproducibility

The data and Supplementary materials associated with this research are available at https://osf.io/fgvma/.
